# Mycotoxin contamination in feeds and feed materials in China in year 2020

**DOI:** 10.3389/fvets.2022.1016528

**Published:** 2022-10-10

**Authors:** Anping Li, Wei Hao, Shu Guan, Jinyong Wang, Gang An

**Affiliations:** ^1^Department of Animal Nutrition and Health, DSM (China) Co., Ltd., Shanghai, China; ^2^Department of Animal Nutrition and Health, DSM Singapore Industrial Pte Ltd., Singapore, Singapore

**Keywords:** mycotoxin contamination, corn, feed, China, co-contamination

## Abstract

A survey of mycotoxin contamination in feed commodities in China was performed and the regional differences of mycotoxin contamination in new season corn was assessed during January 2020–November 2020 in this research. 1,610 samples were analyzed for the major mycotoxins, namely aflatoxins, zearalenone (ZEN), trichothecenes type B, fumonisins (FUM), fusariotoxin T-2 (T-2) and ochratoxin A (OTA) using methods of liquid chromatography—tandem mass spectrometry and enzyme linked immunosorbent assay. Generally, aflatoxins occurred in 16% of all samples, and ZEN, trichothecenes type B and FUM were more prevalent with positive rates of 47, 72, and 63%, respectively. T2 and OTA were rarely detected. In new season corn, samples were also seriously contaminated with ZEN, trichothecenes type B, and FUM at positive rates of 47, 76, and 79%, respectively, and their averages of positives were 112, 735, and 3,811 μg/kg, respectively. The patterns of mycotoxin occurrence showed distinct regional trends in new season corn samples. Samples from Shandong province were highly contaminated with FUM, while special attention should be paid to aflatoxins in Anhui and Jiangsu provinces of East China. The contents of trichothecenes type B and ZEN from northern to southern provinces showed downward trends. In new season corm, co-occurrence of mycotoxins was widespread, and combinations of ZEN, trichothecenes type B, and FUM were frequently observed in this study. Trichothecenes type B and ZEN concentrations showed a positive correlation coefficient of 0.294, suggesting that toxicological interactions of these toxins deserve attention.

## Introduction

Mycotoxins are toxic fugal secondary metabolites and can be frequently found in the livestock industry as contaminants in feeds and feed raw materials. The most commonly discovered mycotoxins are aflatoxins (e.g., aflatoxin B1, AFB1), fumonisins (FUM), zearalenone (ZEN), trichothecenes (type B, e.g., deoxynivalenol, DON; type A, e.g., T-2 toxin, T-2), and ochratoxin A (OTA). When the concentrations of mycotoxins in feeds exceed a certain level, a variety of adverse effects can be caused on animals' health, causing immunotoxicity and impairing the reproductive function in farm animals (1). In addition, exposure of tissues, such as the kidneys, liver, and intestines, to mycotoxins can exert histopathological changes that can interfere with animal growth and survival (2).

There are multiple factors that can affect the contamination of mycotoxins on feeds and raw materials. Different plant species possess different levels of susceptibility to fungal infestation, and environmental conditions, such as temperature and precipitation, can also determine the infestation of mycotoxin-producing fungi and the accumulation of mycotoxins (3).

Crop plants can be infested with multiple strains of fungi, and most fungi strains can produce multiple mycotoxins. Therefore, co-contamination of crop plants with multiple mycotoxins is frequently detected (4), and the multiple mycotoxins usually cause synergistic effects to cause more serious toxic effect on animals' immunity and health (5).

Numerous countries have established maximum levels or guidance values to regulate the mycotoxins contamination in food and feeds. China, as one of the countries with serious mycotoxin problems, has formulated a series of standards on mycotoxins of AFB1, ZEN, DON, FUMs, OTA and T-2 in feeds, for further risk management of mycotoxins. Currently, to support the availability of mycotoxin risk data, annual mycotoxin surveys have been conducted in China to monitor the occurrence of regular mycotoxins in feeds and feed raw materials. Studies on these data have been published previously (6–8). In this study, we analyzed the contamination of aflatoxins, ZEN, trichothecenes type B, T-2, FUM and OTA toxins in 1,610 samples collected in 2020. We compared the pattern of mycotoxin occurrence of different regions in new season corn and analyzed the variation of mycotoxin concentrations in different kinds of feeds and raw materials in China. The aim of this annual survey was to provide the basic information to the mycotoxin contamination of feeds and raw materials in China.

## Materials and methods

### Samples and mycotoxin standards

A total of 1,610 samples of feeds and raw materials were collected from January 2020 to November 2020. The dataset comprised 505 samples of finished feed (124 in swine feed, 270 in poultry feed, 85 in TMR, and 26 in supplementary concentrates), 883 samples of feed raw materials (665 in corn, 72 in wheat, 49 in bran, 63 in soybean meal, 9 in rice bran meal, and 25 in peanut meal), 45 samples of corn by-product [15 in dried distillers' grains and soluble fraction (DDGS), 27 in corn gluten meal and 3 in sprayed corn husk], and 177 samples of grasses (105 in silage, 30 in oat grass, 39 in alfalfa and 3 in soybean hull). The new season corn dataset comprised 214 samples collected from 9 provinces of Heilongjiang, Jilin, Liaoning, Hebei, Shandong, Henan, Gansu, Jiangsu and Anhui provinces in China. Complete notes with details surrounding the circumstances of the samples, including temperature, moisture and water content were submitted with the samples. One kg original samples were collected and kept at 4°C before transported to the analytical Romer Labs in Wuxi, China (8). Sampling, milling and homogenization of a 100 g representative sub-sample were performed as described by Kovalsky et al. (9).

### Mycotoxin analysis

For 903 samples with relatively complex matrices, such as DDGS, finished feed, silage and TMR, a multi-mycotoxin analysis method of liquid chromatography—tandem mass spectrometry (LC-MS/MS) was performed for the analysis of mycotoxin occurrence. Through this method, a total of 18 mycotoxins, including 4 kinds of aflatoxins (AFB1, AFB2, AFG1, AFG2), ZEN, 5 kinds of trichothecenes type B (DON, 3-Acetyl-Deoxynivalenol, 15-Acetyl-Deoxynivalenol, Nivalenol, Fusarenon X), 4 kinds of trichothecenes type A (T-2, HT-2 toxin, Diacetoxyscirpenol, Neosolaniol), 3 kinds of fumonisins (FB1, FB2, FB3) and OTA were detected simultaneously (9). Procedures of analysis and quality control were carried out as described previously (6). Limits of detection (LODs) of this method for each mycotoxin were listed as follows: 0.5 μg/kg for AFB1, 0.5 μg/kg for AFB2, 0.5 μg/kg for AFG1, 0.5 μg/kg for AFG2, 10 μg/kg for ZEN, 10 μg/kg for DON, 10 μg/kg for FB1, 10 μg/kg for FB2, 10 μg/kg for FB3, 0.5 μg/kg for OTA and 10 μg/kg for T-2 toxin, respectively.

For the remaining 707 samples of feed raw materials with relatively simple matrices, such as corn, wheat and bran, samples were detected using the method of enzyme linked immunosorbent assay (ELISA). Procedures of sample preparation and analyses were performed with commercially available test kits (AgraQuant^®^ Assay, Romer Labs Diagnostic GmbH, Austria) according to their operating instructions. LODs were 2 μg/kg for AFB1, 25 μg/kg for ZEN, 250 μg/kg for DON, 250 μg/kg for FUM, 10 μg/kg for T-2, and 2 μg/kg for OTA, respectively.

For all analyzed samples, the threshold of mycotoxin concentration was defined as: >1 μg/kg for the sum of AFB1, AFB2, AFG1 and AFG2, >32 μg/kg for ZEN; >50 μg/kg for trichothecenes type B, >100 μg/kg for FUM, >30 μg/kg for T-2 toxin, and >2 μg/kg for OTA. Correlations between mycotoxin contaminations were analyzed with the ggpairs in the ggally package (10) using R software, version 3.3.0 (11). Results below the LODs were treated as zero values in the correlation analysis.

## Results

### Mycotoxin occurrence in general and in new season corn

In this study, a total of 1,610 samples of feeds and raw materials, including 214 samples of new season corn, were collected throughout China in year 2020. The numbers of mycotoxin tested samples, the respective positive rates, the levels of average positives and maximum with regard to different types of mycotoxins were summarized in Table 1. The maximum level of aflatoxins detected was 482 μg/kg in a corn sample, and the maximum ZEN level was 11,245 μg/kg and detected in a corn gluten meal sample. Trichothecenes type B (72%) and FUM (63%) were highly prevalent among the 6 mycotoxins and the most serious contamination were found in a silage sample and a corn gluten meal sample with the concentrations of 10,426 and 30,872 μg/kg, respectively. T-2 and OTA, on the other hand, were rarely found in 5 and 1% of the samples, respectively. The highest T-2 toxin concentration was sourced in a corn sample with 111 μg/kg, and the highest OTA concentration was detected with concentration of 30 μg/kg in a corn gluten meal sample. The levels of average positives for aflatoxins, ZEN, trichothecenes type B, FUM, OTA and T-2 were 34, 206, 651, 1,570, 7, and 59 μg/kg, respectively.

**Table 1:** Mycotoxin contamination in general and in new season corn in China in year 2020.

**Items**	**Aflatoxins**	**Zearalenone (ZEN)**	**Trichothethenes type B**	**Fumonisins (FUM)**	**Ochratoxin A (OTA)**	**T-2 toxin**
**In general**						
Sample number	1,610	1,610	1,610	1,610	973	1,563
Positive rate	16%	47%	72%	63%	5%	1%
Average of positives (μg/kg)	34	206	651	1,564	7	59
Maximum (μg/kg)	482	11,245	10,426	30,872	30	111
Source of maximum	Corn	Corn gluten meal	Silage	Corn gluten meal	Corn gluten meal	Corn
**In new season corn**						
Sample number	214	214	214	214	17	187
Positive rate	23%	47%	76%	79%	0%	2%
Average of positives (μg/kg)	52	112	735	3,811	0	77
Maximum (μg/kg)	482	1,572	4,670	23,480	0	111
Source of maximum	Jiangsu and Anhui	Hebei	Heilongjiang	Henan	–	Jiangsu and Anhui

In new season corn, the Fusarium mycotoxins ZEN, trichothecenes type B, and FUM were most prevalent and were detected in 47, 76, and 78% of samples, with highest concentrations of 1,572, 4,670, and 23,480 μg/kg, sourced in Hebei, Heilongjiang, and Henan provinces of China. Aflatoxins were detected with the positive ratio of 23%. OTA was not detected, and T-2 toxin was only found in 2% of the detected samples. The averages of positives were 52, 112, 735, and 3,811 μg/kg for mycotoxins of aflatoxins, ZEN, trichothecenes type B, and FUM, respectively.

### Mycotoxin occurrence in different feed raw materials

The occurrence of mycotoxins in different feed materials was presented in Table 2. In corn samples, ZEN (42%), trichothecenes type B (73%) and FUM (62%) occurred more frequently, with the average positive concentrations of 124, 740, and 2,300 μg/kg, and maximum levels of 1,307, 3,930, and 23,480 μg/kg, respectively. Thirteen percentage samples were detected with contamination of aflatoxins with average level of 42 μg/kg in corn. In wheat, ZEN, trichothecenes type B, and FUM were found in 42, 33, and 29% of detected samples. The average of positives was 1,030 μg/kg for trichothecenes type B and the highest level was detected with concentration of 5,700 in one wheat sample. ZEN were prevalent in soybean meal with a high fraction (75%) at a relatively low average of positives of 50 μg/kg. In peanut meal, aflatoxins were found to be positive in all samples and the average of positives was 127 μg/kg, with a relatively high-risk level. Bran samples were mainly contaminated with trichothecenes type B (94%) at a positive average of 768 μg/kg. Rice bran meal was detected to be mainly contaminated with ZEN at positive rate of 100%. All samples of feed raw materials were rarely contaminated with OTA and T-2 toxins.

**Table 2:** Mycotoxin occurrence in feed raw materials.

**Items**	**Aflatoxins**	**Zearalenone (ZEN)**	**Trichothethenes type B**	**Fumonisins (FUM)**	**Ochratoxin A (OTA)**	**T-2 toxin**
**Corn**						
Sample number	665	665	665	665	115	665
Positive rate	13%	42%	73%	62%	4%	1%
Average of positives (μg/kg)	42	124	740	2,300	9	69
Maximum (μg/kg)	482	1,307	3,930	23,480	26	111
**Wheat**						
Sample number	72	72	72	72	72	2
Positive rate	0%	42%	33%	29%	3%	0%
Average of positives (μg/kg)	0	81	1,030	308	4	0
Maximum (μg/kg)	0	116	5,700	710	4	0
**Soybean meal**						
Sample number	63	63	63	63	16	16
Positive rate	14%	75%	0%	0%	6%	0%
Average of positives (μg/kg)	3	50	0	0	2.4	0
Maximum (μg/kg)	6	96	0	41	2.4	0
**Peanut meal**						
Sample number	25	25	25	25	25	25
Positive rate	100%	8%	4%	0%	12%	4%
Average of positives (μg/kg)	127	49	76	–	3	35
Maximum (μg/kg)	217	61	76	54	3	35
**Bran**						
Sample number	49	49	49	49	9	9
Positive rate	14%	4%	92%	2%	14%	4%
Average of positives (μg/kg)	3	83	768	121	6	0
Maximum (μg/kg)	4	84	2,180	121	6	0
**Rice bran meal**						
Sample number	9	9	9	9	9	9
Positive rate	44%	100%	56%	33%	22%	0%
Average of positives (μg/kg)	9	105	305	283	5	0
Maximum (μg/kg)	15	283	431	351	7	0

### Mycotoxin occurrence in different corn by-products

As presented in Table 3, ZEN, trichothecenes type B and FUM were more prevalently detected, and their positive rates were almost 100% in the corn by-products samples in this survey. In DDGS, the positive average of trichothecenes type B was twice as that in the corn samples with the value of 1,536 μg/kg. In corn gluten meal, aflatoxins were detected in 37% of the samples with average of positives of 67 μg/kg, and the positive averages of ZEN and FUM reached 2,743 and 6,562 μg/kg, respectively. In 3 samples of sprayed corn husk, Fusarium toxins were concentrated with average positive values of 494, 4,187, and 3,377 μg/kg, in ZEN, trichothecenes type B, and FUM respectively. All samples of corn by-products were analyzed for OTA and T-2 contamination, and the levels detected were rather low.

**Table 3:** Mycotoxin occurrence in corn by-products.

**Items**	**Aflatoxins**	**Zearalenone (ZEN)**	**Trichothethenes type B**	**Fumonisins (FUM)**	**Ochratoxin A (OTA)**	**T-2 toxin**
**Distillers dried grains with solubles (DDGS)**						
Sample number	15	15	15	15	15	15
Positive rate	7%	100%	100%	87%	40%	0%
Average of positives (μg/kg)	23	115	1,536	1,625	3	0
Maximum (μg/kg)	23	239	3,267	6,160	5	0
**Corn gluten meal**						
Sample number	27	27	27	27	27	27
Positive rate	37%	100%	81%	100%	41%	0%
Average of positives (μg/kg)	67	2,743	383	6,562	14	0
Maximum (μg/kg)	469	11,245	1,866	30,872	30	0
**Sprayed corn husk**						
Sample number	3	3	3	3	3	3
Positive rate	0%	100%	100%	100%	0%	0%
Average of positives (μg/kg)	0	494	4,187	3,377	0	0
Maximum (μg/kg)	0	727	6,753	7,062	0	0

### Mycotoxin occurrence in different grasses

As exhibited in Table 4, aflatoxins, OTA and T-2 toxins showed negative in all samples of silages and grasses, expect one sample of silage with aflatoxins concentration of 21 μg/kg, and one sample of oat with T-2 concentration of 53 μg/kg. In silages, ZEN, trichothecenes type B, and FUM showed positive rates of 42, 75, and 73%, respectively, with average of positives of 123, 1,114, and 510 μg/kg, respectively. Alfalfa samples were only found with contamination of ZEN with positive rates of 8% and positive average of 45 μg/kg. In oat grass, relatively low contamination level of ZEN, trichothecenes type B and FUM were found with average of positives of 144, 159, and 212 μg/kg, respectively. In 3 samples of soybean husk, one was found positive in trichothecenes, and another one is co-contaminated with ZEN and DON. The contamination levels were relatively low.

**Table 4:** Mycotoxin occurrence in grasses.

**Items**	**Aflatoxins**	**Zearalenone (ZEN)**	**Trichothethenes type B**	**Fumonisins (FUM)**	**Ochratoxin A (OTA)**	**T-2 toxin**
**Silages**						
Sample number	105	105	105	105	105	105
Positive rate	1%	42%	75%	73%	0%	0%
Average of positives (μg/kg)	21	123	1,114	510	0	0
Maximum (μg/kg)	21	654	10,426	2,473	0	0
**Alfalfa**						
Sample number	39	39	39	39	39	39
Positive rate	0%	8%	0%	0%	0%	0%
Average of positives (μg/kg)	0	45	0	0	0	0
Maximum (μg/kg)	0	82	0	0	0	0
**Oat grass**						
Sample number	30	30	30	30	30	30
Positive rate	0%	13%	10%	3%	0%	3%
Average of positives (μg/kg)	0	144	159	212	0	53
Maximum (μg/kg)	0	288	298	212	0	53
**Soybean husk**						
Sample number	3	3	3	3	3	3
Positive rate	0%	67%	33%	0%	0%	0%
Average of positives (μg/kg)	0	69	151	0	0	0
Maximum (μg/kg)	0	79	151	0	0	0

### Mycotoxin occurrence in different finished feeds

Mycotoxin contamination in swine and poultry finished feeds was detected with similar patterns in this study (Table 5). 28 and 22% of samples were detected to be contaminated with aflatoxins, respectively, and ZEN infested more than 50% of samples with positive averages of 123 and 73 μg/kg, respectively, in poultry and swine finished feeds. Trichothecenes type B and FUM showed relatively high prevalence. Concentrations of trichothecenes type B reached positive averages of above 400 μg/kg, and FUM were detected at average concentrations of positives of 990 and 871 μg/kg in poultry and swine feeds, respectively. OTA and T-2 in finished feeds were either detected at rather low levels or not detected in this survey. In samples of cow supplementary concentrate, aflatoxins, ZEN, trichothecenes type B, FUM, and OTA were detected with positive rates of 8, 65, 92, 58, and 4%, at average concentrations of positives of 1, 102, 475, 400, and 2 μg/kg, respectively. In TMR, positive samples of ZEN, trichothecenes type B, FUM were at levels of 68, 96, and 88% with average concentration of 111, 456, and 370 μg/kg, respectively, and aflatoxins, OTA and T-2 were detected at relatively low mycotoxin risk levels.

**Table 5:** Mycotoxin occurrence in finished feeds.

**Items**	**Aflatoxins**	**Zearalenone (ZEN)**	**Trichothethenes type B**	**Fumonisins (FUM)**	**Ochratoxin A (OTA)**	**T-2 toxin**
**Poultry feed**						
Sample number	270	270	270	270	270	270
Positive rate	28%	56%	96%	91%	5%	0%
Average of positives (μg/kg)	7	123	409	990	5	0
Maximum (μg/kg)	51	1,094	1,173	4,136	27	0
**Swine feed**						
Sample number	124	124	124	124	124	124
Positive rate	22%	51%	95%	95%	5%	1%
Average of positives (μg/kg)	9	73	442	871	4	33
Maximum (μg/kg)	85	331	3,443	4,511	5.4	33
**Total mixed ration (TMR)**						
Sample number	85	85	85	85	85	85
Positive rate	1%	68%	96%	88%	0%	0%
Average of positives (μg/kg)	3	111	456	370	0	0
Maximum (μg/kg)	3	719	3,047	1,287	0	0
**Supplementary concentrate**						
Sample number	26	26	26	26	26	26
Positive rate	8%	65%	92%	58%	4%	0%
Average of positives (μg/kg)	1	102	475	400	2	0
Maximum (μg/kg)	2	717	1,241	1,321	2	0

### Mycotoxin occurrence of new season corn in different regions of China

The regional patterns of mycotoxin contamination of new season corn were elucidated in Table 6. The whole dataset of corn samples was decomposed into the sub-datasets of 5 geographical regions including 9 major corn-producing provinces in China, i.e., Heilongjiang, Jilin, Liaoning provinces of Northeast China, Hebei province of North China, Henan province of Central China, Shandong, Jiangsu and Anhui provinces of East China, and Gansu province of Northwest China.

**Table 6:** Mycotoxin occurrence in new season corn in different regions of China.

**Items**	**Aflatoxins**	**Zearalenone (ZEN)**	**Trichothethenes type B**	**Fumonisins (FUM)**	**Ochratoxin A (OTA)**	**T-2 toxin**
**Northeast China**						
Sample number	68	68	68	68	8	41
Positive rate	0%	56%	96%	57%	0%	0%
Average of positives (μg/kg)	0	124	934	1,651	0	0
Maximum (μg/kg)	0	486	4,670	7,073	0	0
**North China**						
Sample number	25	25	25	25	0	25
Positive rate	4%	68%	88%	88%	–	0%
Average of positives (μg/kg)	2	169	876	2,630	–	0
Maximum (μg/kg)	2	1,572	1,950	7,000	–	0
**East China**						
Sample number	54	54	54	54	4	54
Positive rate	33%	37%	61%	93%	0%	6%
Average of positives (μg/kg)	100	107	563	5,204	0	70
Maximum (μg/kg)	482	592	1,660	21,620	0	111
**Central China**						
Sample number	60	60	60	60	5	60
Positive rate	52%	37%	67%	85%	0%	0%
Average of positives (μg/kg)	25	60	371	4,872	0	–
Maximum (μg/kg)	265	160	1,090	23,480	0	0
**Northwest China**						
Sample number	7	7	7	7	0	7
Positive rate	0%	57%	43%	71%	–	14%
Average of positives (μg/kg)	0	78	2,123	1,112	–	97
Maximum (μg/kg)	0	113	3,930	2,420	–	97

For Northeast China, aflatoxins, OTA and T-2 toxins were not detected. Trichothecenes Type B was found frequently (positive rate 96%) with positive average of 934 μg/kg. FUM were detected with positive rate of 57% and positive average of 1,651 μg, and the contamination level of ZEN was detected with the positive rate and average of 56% and 124 μg/kg, respectively.

For North China, aflatoxins were detected in 4% of samples at a positive average of 2 μg/kg. 68, 88, and 88% of the samples were found to be contaminated with ZEN, trichothecenes type B, and FUM, and their positive average concentrations were 169, 876, and 2,630 μg/kg, respectively. OTA and T-2 were not detected.

Aflatoxins were detected in 33% of samples from East China, and the average of positives was 100 μg/kg, which was the highest median concentration obtained for any region. The positive rate of ZEN was 37%, and trichothecenes type B and FUM were dominating with prevalence of 61 and 93%, and positive averages of 563 and 5,204 μg/kg, respectively. Six percentage of samples from east China were positive at T-2 with positive average of 70 μg/kg in this survey.

For Central China, aflatoxins were detected with relatively high positive proportion of 52%, and average concentration of 47 μg/kg. Trichothecenes type B and FUM were detected in 67 and 85% of the samples, respectively, and the positive average of FUM reached as high as 4,872 μg/kg. ZEN was detected in 37% of the samples.

In the 7 samples from Northwest China, occurrence of ZEN, trichothecenes type B, FUM and T-2 in samples from this region was 57, 43, 71, and 14% respectively. For trichothecenes type B, the highest level detected was 3,930 μg/kg and the positive average was 2,123 μg/kg. ZEN, FUM and T-2 were positive with the average levels of 78, 1,112, and 97 μg/kg, respectively, in this region.

### Co-occurrence of mycotoxins in new season corn

**Figure 1:**
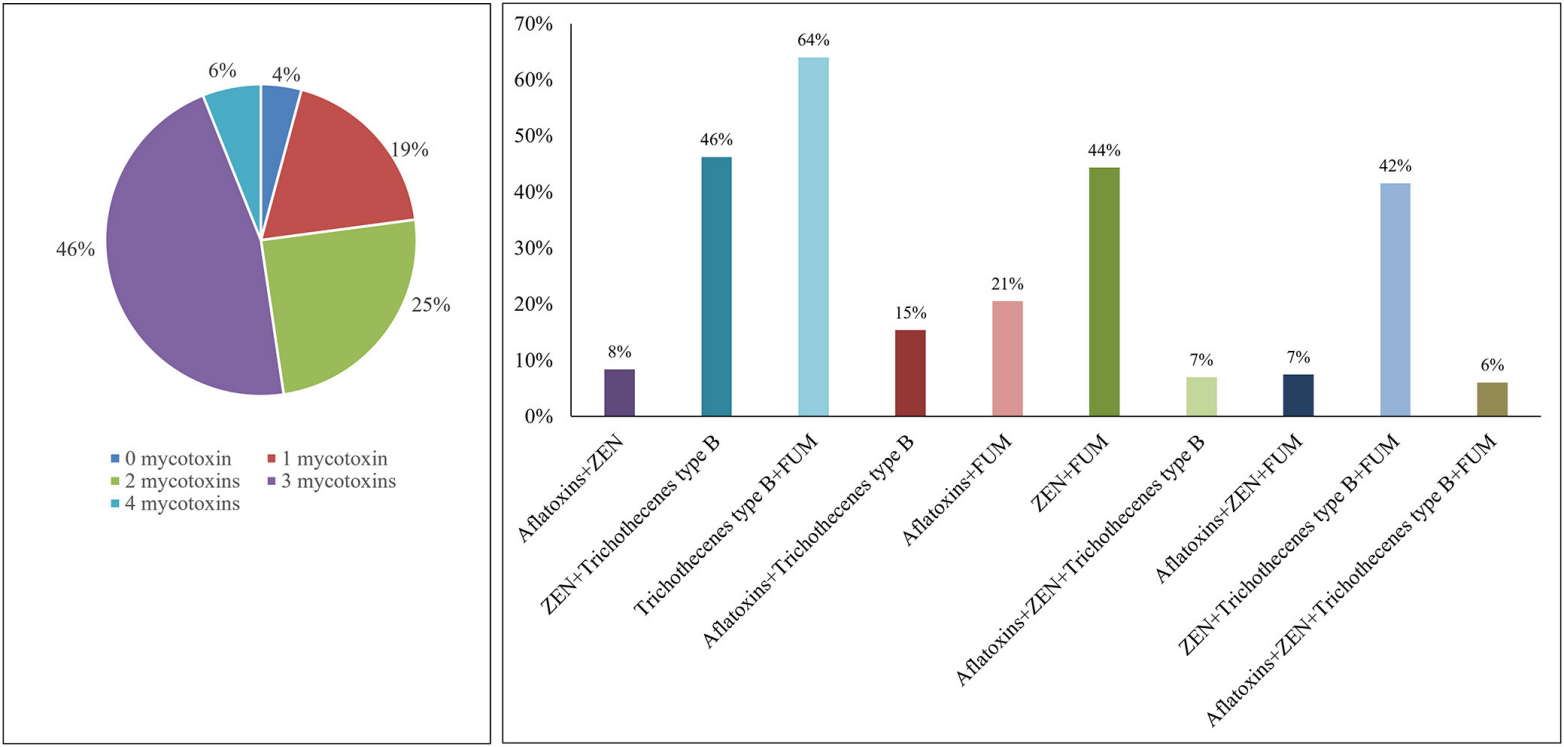
Mycotoxin co-occurrence in new season corn.

To analyze the co-occurrence of mycotoxins in new season corn, the fractions of samples contaminated with either combination of two to four mycotoxins were calculated in this study (Figure 1). Since OTA and T-2 were not evaluated in all samples, this co-occurrence ratio was only calculated in the most common four mycotoxins in new season corn samples, that are aflatoxins, ZEN, trichothecenes type B and FUM. Most of the samples contained multiple mycotoxins with 2–3 classes (71%), and 6% of the samples were detected with contamination with all 4 mycotoxins. The combination of trichothecenes type B and FUM was most frequently observed, with the proportion of 64%. ZEN and trichothecenes type B, and ZEN and FUM co-occurred in 46 and 44%, respectively. Three mycotoxins combination of ZEN, trichothecenes type B and FUM was detected in 42% of the samples.

**Figure 2:**
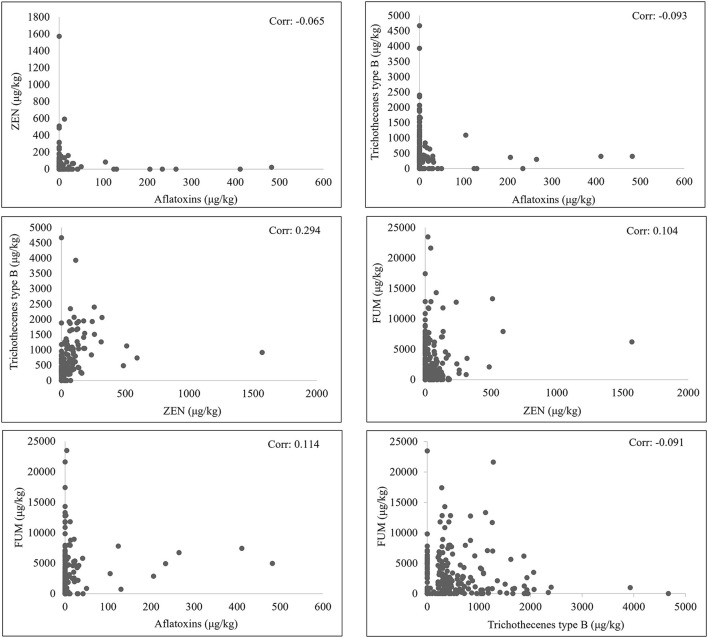
Correlation of mycotoxin concentrations of new season corn.

In Figure 2, any combination of two mycotoxins in the samples of new season corn were presented. A positive correlation of trichothecenes type B and ZEN concentrations was showed with a coefficient of 0.294. While other combinations showed correlation coefficients of either <0.2 or negative.

## Discussion

Mycotoxins have been proved to reduce animal performance, threaten human health and bring huge economic loss to feed and food industry each year (1, 12). Considering the widespread contamination, aflatoxins, ZEN, trichothecenes type A represented by T-2, trichothecenes type B represented by DON, FUM, and OTA are among the most important and dominating mycotoxins. China, as one of the countries with serious mycotoxin pollution, has placed stricter regulations on mycotoxins in feeds and raw materials. However, surveys of mycotoxin contamination on certain feed commodities in China are limited. Thus, we conducted a series of survey to monitor the mycotoxin contamination and this study focused on the occurrence of the six major mycotoxins in feeds from different regions of China in year 2020.

In general, aflatoxins showed a relatively low prevalence in feeds and the levels of positive samples were mostly unthreatening for most of the samples complied with the current regulatory restrictions in China. Similar results were also obtained previously. In a global survey of mycotoxin contamination performed for 10 years from 2008 to 2017, aflatoxins occurrence in East Asia were detected with a positive rate of 17.1% and a median of positives of 10 μg/kg (13). In another survey focused on China, the result of AFB1 contamination was also similar while AFB1 showed a relatively higher positive rate at 38.2% probably due to the climatic conditions of that year or the different source of collected samples (8). Aflatoxins were tested to be positive in all samples of peanut meal in this study, and the 100% positive rate was also found in the survey of year 2021 (6). These results should draw attention because aflatoxins synthetizing strains, *Aspergillus flavus* and *A. parasiticus* are particularly easily to infest corn and nuts according to previous studies (14).

Fusarium toxins dominated in the mycotoxin profiles in feed materials and products in this report. The occurrence of ZEN, trichothecenes type B and FUM in all samples ranged from 47 to 72%. Their levels were comparable in previous studies, and for the samples from East Asia, the positive rates of ZEN, trichothecenes type B and FUM were 58, 85, and 61%, respectively, with median concentrations of 90, 418, and 810 μg/kg, respectively (13). Corn is the most frequently used feed materials in China. The corn samples, in this research and in other previously published reports, showed high prevalence and high levels of FUM, DON, and ZEN (8, 15). Wheat samples were also mainly polluted by DON and additionally contained ZEN, and in rice, ZEN and DON were most frequently detected. These results are predictable because some certain fungal species have been well-proved to have associations with the fungal infestation of specific crops. For example, corn is prone to be polluted by FUM producer *F. verticillioides*, and wheat, rice, and barley can be easily infested by strains of *Fusarium culmorum* and *F. graminearum* which are well-known as DON and ZEN producers (16, 17).

Patterns of mycotoxin contamination was varied from region to region and year to year, and the variation could be traced back to the variation of weather conditions.

Trichothecenes type B showed relatively high levels in the northeast and north regions of China, and their content in samples from northern to southern provinces of China showed a significant downward trend. This result was consistent with previous studies in which higher DON concentrations were detected in samples from the temperate regions, such as North America, Northern Europe, Central Europe, and East Asia (13). Mild temperatures and rainfall during the flowering and maturation periods would facilitate the infestation of corn with strains of *F. graminearum* and *F. culmorum* and subsequent DON contamination (18). Therefore, the high level of trichothecenes type B in Northeast China could be correlated with the increased rainfall caused by the attach of typhoons in August and September. The positive average of trichothecenes type B in Gansu province of Northwest China was also at extremely high level of 2,123.33 μg/kg in 2020, and this could be due to the higher than usual rainfall in August in Gansu province. ZEN was observed to frequently co-contaminate corn with DON (4, 13, 19), and ZEN also showed a medium-high risk level in the northeast, north and east regions of China in this study. Which should be noted here is that the contamination levels of trichothecenes type B and ZEN were significantly increased in new season corn harvested in year 2021, especially in Shandong province of East China (6). These peaks corresponded with climate conditions of higher than usual rainfall and more frequent typhoon attacked in July and August in Shandong province, which furtherly confirmed the key impact of rainfall on DON and ZEN contamination levels.

FUM contaminations in East (Shandong, Anhui and Jiangsu provinces) and Central (Henan) China were significantly higher than in other regions surveyed in this study. Anhui, Jiangsu and Henan provinces showed relatively hot temperatures in summer season of 2020, and high temperature and low precipitation during the corn silking period could contribute to the *F. verticillioides* infestation and aggravate the pollution of FUM (20, 21). When compared to year 2020, FUM concentration was significantly higher in corn harvested in 2021, and this variation was magnified in Jilin province which is regarded as the main corn producing province in China (6). Considering the weather conditions, warmer temperature and the consequent lower amount of rainfall occurred in July and August, and this could contribute to the increased FUM contamination in Jilin province of that year.

The prevalence and pollution level of aflatoxins were significantly higher in samples from Jiangsu and Anhui provinces than other regions of China, and the positive values were found to be higher than in year 2021 (6). It has been well-demonstrated that *Aspergillus* spp. infestation and aflatoxins production can be favored by relatively high temperature and low humidity during the corn growing period (22), and the weather conditions feathering heavy precipitation in hot season of this region may facilitate the prevalence of aflatoxins. Especially in Anhui province, the phased drought condition occurred in early summer, followed by relatively heavier rainfall during the corn growing and harvest periods. It's these unusual weather conditions in Anhui province that might lead to a significant increasing level of aflatoxins contamination in year 2020. Moreover, in addition to climate conditions, timing of harvest, post-harvest handling and storage of feed raw materials may also have significant effects on the formation and accumulation of mycotoxins. This is worth noting for the feed safety.

As exhibited in this survey, trichothecenes type B and ZEN can be highly correlated in corn, especially in the temperate regions. This result has been widely found and can be expected because both mycotoxins can be produced by strains of *F. graminearum* and *F. culmorum* (8, 9, 13). Even though the exposure of animals to DON and ZEN are common, serious attention should be paid for the safe of animal health. When mycotoxins co-occur, their combined toxic effects, mainly additive or synergistic effects have been observed on different parameters of function in different species of animals. The immune function (23), the intestinal barrier function (24, 25), the liver health (26), the oxidative stress in spleen (23), brain (27) and kidneys (28) can all be affected with different levels in mice and pigs. The co-occurrence and interactions between other frequently detected mycotoxins were also regularly found (8, 13, 29). Results suggested that co-contaminates, such as aflatoxins and trichothecenes, ochratoxins and FUMs, could exhibit stronger toxic effects on animals when compared to each individual mycotoxin (30, 31).

In this study, it is very common to detect the co-contamination of trichothecenes type B, ZEN and FUM or aflatoxins and FUM in samples of new season corn. Considering finished feeds are commodities of different blended feed raw materials and then should contain a combination of mycotoxins, finished feeds can be expected to show a more complicated situation of co-contaminates. As a considerable number of samples are usually found to contain more than one mycotoxins, constant monitoring and continued research effort on prevention and mitigation of co-contamination of mycotoxin are therefore desperately necessary. Moreover, in case the negative effects of certain feeds and materials can be underestimated, the further investigation of the possible additive or synergistic effects of co-occurring mycotoxins on animals would be important to clarify. Since the current regulations on mycotoxins concentrations in China have not considered the co-contamination of mycotoxins the researches focusing on the toxicity of mycotoxin combinations are important.

Considering the annal climate parameters of 2020, due to the frequent extreme weather conditions, such as typhoons, the risk of mycotoxin contamination of raw materials and feed products should draw attention. In new season corn, ZEN, trichothecenes type B, and FUM were the most serious and dominating mycotoxins determined, and their contamination levels were also extremely high in finished feed products. Although China has established strict control regulations, there are also some samples tested with high concentrations of mycotoxins on the market. Since mycotoxins show both health and economic implications on animals and consumers, strict strategies of monitoring and supervision should be continued, and effective measures of prevention and control should be established. Application of targeted detoxification products is considered to be the most effective, safe and environmentally friendly measure to control the contamination of mycotoxins. In this way, the mycotoxin contamination at the sources of feed and food production can be minimized and even eliminated.

## Data availability statement

The original contributions presented in the study are included in the article/supplementary material, further inquiries can be directed to the corresponding author/s.

## Author contributions

AL: data analysis, investigation, and writing – original draft. WH: conceptualization, data analysis, validation, writing – original draft, and editing. SG: writing – review, editing, supervision, and project administration. JW: validation, investigation, and resources. GA: supervision, project administration, and funding acquisition. All authors contributed to the article and approved the submitted version.

## Conflict of interest

AL, WH, SG, JW, and GA were employed by DSM China Co., Ltd, China and DSM Singapore Industrial Pte Ltd.

## Publisher's note

All claims expressed in this article are solely those of the authors and do not necessarily represent those of their affiliated organizations, or those of the publisher, the editors and the reviewers. Any product that may be evaluated in this article, or claim that may be made by its manufacturer, is not guaranteed or endorsed by the publisher.
